# A crosstalk between tumor cells and adipocytes facilitates tumor cell migration and invasion

**DOI:** 10.7150/ijbs.117604

**Published:** 2026-03-04

**Authors:** Laura Garrido-Jiménez, Beatriz del Valle-Pérez, Javier Pastor, Élida Alechaga, Aleix Sala-Vila, Óscar J. Pozo, Raúl Peña, Antonio García de Herreros

**Affiliations:** 1 Cancer Research Program, Hospital del Mar Research Institute, Unidad Asociada al CSIC, Barcelona, Spain.; 2 Department of Medicine and Life Sciences, Universitat Pompeu Fabra, Barcelona, Spain.; 3 Applied Metabolomics Research Group, Hospital del Mar Research Institute, Barcelona, Spain.; 4 Cardiovascular Risk and Nutrition Research Group, Hospital del Mar Research Institute, Barcelona, Spain.

**Keywords:** cancer-associated adipocytes, tumor cell migration, reactive oxygen species

## Abstract

Cancer cell invasion is modulated by their interaction with the tumor microenvironment (TME). In this article we have analyzed the cooperation of one of the TME cellular components, adipocytes, and breast tumor cells. Co-culture of these two types of cells increase tumor cell invasion and migration. This effect is associated to the de-differentiation of adipocytes that lose lipids and experience a transition to a mesenchymal phenotype. Furthermore, tumor cells are activated by adipocytes and undergo a partial epithelial-to-mesenchymal transition (EMT), which is characterized by a slow upregulation of Snail1. While partial EMT and increased migration both require fatty acid internalization, the adipocyte effect in our system does not rely on direct fatty acid transfer; instead, the tumor cells take these compounds directly from the culture medium. Moreover, adipocytes stimulate tumor cell metabolism by increasing glucose consumption and the production of reactive oxygen species (ROS); this metabolic shift is associated with the upregulated expression of NADPH oxidases (NOX) 1 and 5. Accordingly, a NOX inhibitor or NOX1 down-regulation prevents adipocyte-enhanced ROS generation, Snail1 expression and tumor cell migration. These results show that a bidirectional crosstalk between the two types of cells drives adipocyte dedifferentiation and tumor cell migration and invasion.

## Introduction

Breast cancer is the most prevalent neoplasia among women worldwide 1. Epidemiological evidences have associated obesity with increased incidence, progression and metastasis of many human cancers including breast tumors 2-3. Obesity is characterized by a higher number and size of adipocytes; therefore, these cells have been proposed to contribute to breast cancer progression 2-3. Adipocytes are key constituents of the tumor microenvironment in breast cancer and other malignancies. In these cancers, adipocytes situated in close proximity to tumor cells exhibit marked changes in both their phenotype and functionality and, therefore, have been named as cancer-associated adipocytes (CAAs) 4. Histological examinations of solid tumors consistently reveal a reduction in both the number and the size of adipocytes found at the tumor's invasive edge when compared to adipocytes situated in farther regions from the tumor 5.

CAAs display distinct characteristics compared to normal adipocytes, for instance, reduced lipid content, decreased expression of adipocyte markers, and increased synthesis of cytokines 6-7. For instance, CAAs present lower hormone-sensitive lipase, adiponectin and resistin, and also CEBPα (CCAAT/enhancer-binding protein alpha) and PPARɣ (peroxisome proliferator-activated receptor), associated to a decrease in the number and size of lipid droplets 7-8. Moreover, CAAs also show an activated phenotype characterized by an increase expression of pro-inflammatory cytokines such as IL-6 (interleukin-6), IL-1 (interleukin-1) and TNFα (tumor suppressive factor alpha), as well as proteins involved in extracellular matrix remodeling, like matrix metalloproteinase 11 6, 9. Adipocyte activation can be triggered by co-culture with tumor cells. Several growth factors and cytokines have been proposed to mediate this effect 8, 10; it is likely that the precise factor might depend on the specific tumor cell 11. Another factor that might contribute to adipocyte dedifferentiation is the acidification in tumor microenvironment; it has been reported that a decrease in the pH of culture medium promotes lipolysis 12.

CAAs also contribute to tumor invasion. This has been attributed to the release of free fatty acids that are taken up by cancer cells and used by cancer cells for energy production, cell membrane formation, and the synthesis of lipid bioactive molecules 8, 11, 13-14. Moreover, CAAs secrete a variety of metabolites, hormones and cytokines, and some of them have been identified as contributors to tumor progression.

In this work we have set a co-culture of adipocytes and breast tumor cells and analyzed the requirements for adipocytes-stimulated breast tumor migration. We described how a cross-talk between both types of cells is established and show that adipocytes dedifferentiate in presence of tumor cells to facilitate tumor cell activation and migration.

## Materials and Methods

**Cells and Reagents.** AT3 and BTE136, both cell lines derived from MMTV-PyMT murine tumors 15, 16, and the human mammary adenocarcinoma cell lines MCF7 and SKBR3 were grown in Dulbecco's modified Eagle's medium (DMEM, Invitrogen) supplemented with 4.5 g/l of glucose, 100 U/ml penicillin, 100 μg/ml streptomycin, 2 mM glutamine, and 10% fetal bovine serum (FBS) (all from Gibco). All cell lines were maintained at 37°C in a humidified with 5% CO_2_ and were periodically tested to verify that they remained mycoplasma-free. 3T3L1 were kindly provided by Dr. Antonio Zorzano (IRB Barcelona) and differentiated to adipocytes following a standard procedure on gelatin-coated plates. Cells were plated at a concentration of 2.5 x 10^5^ cells per well, in 6-well plates, or 3 x 10^4^ cells per well, in 24-well plates, and cultured for a day. Differentiation was induced by the addition of DMEM (4.5 g/l of glucose) supplemented with 10% FBS, 0.5 mM isobutyl-methyl-xanthine (I5879, Sigma-Aldrich), 0.2 µM dexamethasone (D4902, Sigma-Aldrich) and 10 μg/ml insulin (I1882, Sigma-Aldrich). The differentiation medium was added when cells reached confluence and replenished every three days for a period of 10-12 days.

Cell transfection and selection of transfectants. Murine shRNAs for Nox1 were obtained from Sigma (St. Louis, MO, USA): shNox1.2, TRCN0000046084 (5'-CCGCACACTGA GAAAGCAATT-3'), shNox1.3, TRCN0000046085 (5'-CCAAGGTTGTTATGCACCCAT-3'), and shNox1.4, TRCN0000046085 (5'-GAACAGGAGATGGAGGAATTA-3'). For stable expression, AT3 cells were infected with lentiviral particles containing the shRNA. Infected cells were selected with puromycin at 1μg/ml. Control cells were infected with lentivirus bearing a non-targeting shRNA (clone SHC016; Sigma). Downregulation of the Nox1 was analyzed by RT-PCR. Studies on the effect of Nox1 depletion were performed with shNox1 (1.4) that produced the most consistent effect on Nox1 levels.

Other reagents used in this study were: Atglistatin (Sigma-Aldrich, SML1075), Etomoxir (Selleckchem, 236020), BMS-309403 (MedChemExpress, HY-101903), NOX inhibitor diphenyleneiodonium (D2926, Sigma-Aldrich), LIF (Millipore, ESG1107), IL6 (Peprotech, 216-16), TGFβ (Peprotech, 100-21), N-acetyl cysteine (NAC, Sigma-Aldrich, A9165), Tocopherol (Sigma-Aldrich, T3251), and Trolox (Thermo, 10454155). Fatty acids myristic acid (14:0, ref M3128-10G), pentadecanoic acid (15:0, ref W433400), palmitic acid (16:0, ref P9677), palmitoleic acid (16:1n7, ref P9417), heptadecanoic acid (17:0, ref H3500) and oleic acid (18:1n9, ref O1008) were purchased from Sigma-Aldrich.

**Lipid staining**. Neutral lipids staining was performed with Oil Red O (ORO) or BODIPY staining. For ORO staining, culture medium was removed, cells were washed twice with cold PBS and fixed with 4% paraformaldehyde for 15 min at room temperature. Then PFA was removed and cells were washed twice with cold PBS and a filtered solution 60% Oil Red O (01391, Sigma-Aldrich) was added for 15 min. Cells were washed with cold PBS until Oil Red O solution was completely removed and random pictures were taken using an Inverted Microscope AxioVert A1 (491237-0002-000, Carl Zeiss). The ORO stained area was determined using ImageJ software. Red color was selected by using Color Threshold tool (Hue 20-235, pass selected), and the percentage of selected area was obtained for each image. Alternatively, BODIPY 493/503 (D3922, Invitrogen) was added to a final concentration of 1 µg/ml in PBS for 10 min at 37°C. Random pictures were taken using an Inverted Microscope AxioVert A1 (491237-0002-000, Carl Zeiss) with the appropriate fluorescence filter and lipid droplet positive and lipid droplet negative cells were counted using Qupath software.

**Adipocyte-tumor cells coculture**. Approximately 5 x 10^5^ 3T3L1 cells were differentiated in 6-well plates. After 10-12 days of differentiation, 7 x 10^5^ tumor cells (AT3, BTE136 or MCF7 cells) were seeded on top of 8 µm Transwells (782746, BRAND) in DMEM (10% FBS) for three days. Alternatively, tumor cells were treated with conditioned media of adipocytes. Adipocyte conditioned media (CM) was obtained from differentiated 3T3L1 cell line. After 10-12 days, differentiation media was removed, cells were washed once with PBS and DMEM plus 1% FBS was added; CM was recovered after 48 hours. Tumor cells CM was prepared in the same way from 5 x 10^5^ (80% confluent) cells. CM was used fresh or stored at -80°C until use.

**Determination of palmitic acid uptake by adipocytes and tumor cells**. The determination of ^13^C-labelled palmitic acid was performed following a previously published method 17. Briefly, cells were labelled by incubation with 250 μM ^13^C-labelled palmitic acid (^13^C16, MedChem Express, HY-N0830S6) in regular culture medium during three days. In some specific experiments ^13^C-palmitic acid-labelled adipocytes were incubated with MCF7 cells for three additional days and presence of this labelled fatty acid was determined in both cell extracts. Cells were washed twice with PBS and cell extracts were prepared in sodium dodecyl sulfate (SDS) lysis buffer (50 mM Tris pH 7.5, 10% Glycerol, 2% SDS) (approximately 5 x 10^5^ cell in 200 μl of buffer). Protein concentration was determined and an aliquot of 50 μl of the extract was derivatized using 300 μl of a freshly prepared mixture of o-benzylhydroxylamine, 1 M in acetonitrile:water (2:1) and N-(3- dimethylaminopropyl)-N′-ethylcarbodiimide 1 M in water:pyridine:HCl, (16:1.6:1; pH 5.0). The reaction was conducted at room temperature for 1 hour, under continuous gentle mixing. Then, after addition of 1 ml of ultrapure water, a liquid-liquid extraction was performed with 4 ml of ethyl acetate. The organic layer containing the derivatized ^13^C-labelled palmitic acid was dried under a nitrogen stream (< 15 psi) in a 40ºC water bath. Finally, the extracts were reconstituted with 200 µl of pure methanol and transferred to liquid chromatography-mass spectrometry vials for analysis. For quantitation of ^13^C-labelled palmitic acid, calibration standards at 0, 100, 250, 500 and 1000 ng/ml were prepared following the same procedure and analyzed with the culture samples.

Liquid Chromatography with tandem mass spectrometry (LC-MS/MS) analysis was performed on an Acquity I-Class UPLC system (Waters Associates) coupled to a triple quadrupole (Xevo TQ-S micro) mass spectrometer with an orthogonal Z-spray-electrospray interface (ESI) (Waters Associates, Milford, MA, USA). Nitrogen 99.99% was used as drying and nebulizing gas, and argon 5.0 as collision gas. Source was kept at 150ºC and the desolvation gas was set at a flowrate of 1200 l/h and a temperature of 600ºC, whereas the cone gas was set at 50 l/h. Positive ionization mode was used with a capillary voltage of 3 kV. The chromatographic separation was carried out on an Acquity BEH C18 column (Waters Associates, 2.1x100 mm, 1.7 µm) with a mobile phase composed of a mixture of methanol and water, both with 1 mM ammonium formate and 0.01% formic acid. The injection volume was 10 µl. The following selective transitions for both labelled and unlabeled palmitic acid were acquired by a Selected Reaction Monitoring (SRM) method: 378>124 and 378>91 for ^13^C_16_-palmitic acid, and 362>124 and 362>91 for natural palmitic acid. These SRM transitions allowed the detection of ^13^C-labelled palmitic acid without any interference from the natural palmitic acid present in the samples, regardless of their concentration. Calibration curves for ^13^C_16_-palmitic acid were constructed by 1/x weighted least squares linear regression of the peak areas of labelled palmitic acid. MassLynx software V4.1 and TargetLynx XS were used for data acquisition and processing.

**Total RNA-sequencing**. RNA was obtained from differentiated 3T3L1 and MCF7 co-cultured in Transwells and quantified as indicated in Suppl Methods. RNA integrity was estimated by using RNA 6000 Nano Kit (NC1783726, Agilent) and ADNds Qubit (Invitrogen). RNA amount oscillated between 1.978 µg and 3.104 µg and RIN values between 8.7 and 10. RNA-seq libraries were prepared with mRNA-Seq Illumina Platforms Kit (Roche) following the manufacturer's recommendations. The libraries were sequenced on a NovaSeq 6000 S1 (Illumina) with a read length of 2×50 bp. 6000 SBS kit (Illumina) obtaining >30M PE reads/sample. Sequencing quality control was assessed using the manufacturer's software (Illumina Sequencing Analysis Viewer). Differential expression was analyzed by Limma Voom. A complete list of the genes induced in the different conditions has been deposited in NCBI's Gene Expression Omnibus (GEO) (GSE273442, token ahmfwawajjulhov). Gene set enrichment analysis was performed with GSEA (v4.1.0) software against April 2021 datasets. Datasets were considered statistically positive when FDR > 25% and P value < 0.05.

**SEAHORSE determinations**. Approximately 4 x 10^4^ MCF7 cells were plated on a Seahorse plate in control or treated conditions (adipocyte conditioned media). After 48 hours, cells were washed twice with Seahorse XF DMEM medium (Agilent) supplemented with 5 mM Hepes pH 7.6, 10 mM glucose, 100 mM sodium pyruvate, and 2 mM glutamine (Thermo Fisher Scientific). Then, cells were incubated in the same media for 1 h at 37ºC without CO_2_. Oxygen consumption and ECARs were simultaneously recorded by a Seahorse XFe24 analyzer (Agilent) using the Mito Stress Test protocol, in which cells were sequentially perturbed with 1.5 μM Oligomycin, 3 μM Carbonyl cyanide-4 (trifluoromethoxy) phenylhydrazone (FCCP), 1 μM Antimycin and 1 μM Rotenone (all from Sigma). Data were analyzed using the Seahorse Wave Desktop Software (v.2.6.3, Agilent). Data was normalized by protein concentration obtained with the BSA protein assay.

**Glucose quantification**. Measurement of the glucose concentration of the media was performed using the Glucose Assay Kit (Abcam, ab65333) according to the manufacturer's protocol. The fluorescence was measured at Ex/Em= 535/587 nm using a microplate reader (Tecan). Data was normalized by cell number.

**Reactive oxygen species (ROS) determination**. Approximately 6 x10^4^ cells were cultured on a white p96- well plate and ROS was measured using a cellular reactive oxygen species assay kit (Abcam, ab113851). Briefly, cells were labelled with DCFDA (2',7'-dichlorofluorescin diacetate), a redox sensitive fluorescent probe that it is oxidized by hydroperoxyl, peroxyl, and other ROS species. Cells were incubated for 1h at 37ºC in the dark and fluorescence was measured at different time points. The fluorescence was measured at Ex/Em = 485/535 nm using a microplate reader. Data was normalized by cell number or total protein.

**Tumor bioinformatic analysis**. A heatmap of differentiated 3T3L1 (d3T3L1) adipocytes versus d3T3L1 + MCF7 mature adipocyte markers (Adipoq, Plin1, Fabp4), adipogenic master regulators and early differentiation markers (Pparg, Cebpa, Lpl), mesenchymal stem cell or adipocyte progenitor genes (Cd44, Ly6a, Cd24a, Cd9), macrophage markers (Lgals3), and fibroblast genes (Acta2, Vim, Serpinh1) expression was generated in RStudio with the GSVA package 18. The list of genes and markers used was obtained from Zhu *et al.* 14.

## Results

### Adipocytes dedifferentiate when cultured with tumor cells

To study the adipocyte-tumor cell functional interaction we co-cultured both types of cells. We used 3T3-L1 cells that, under the appropriate conditions, differentiate to adipocytes, as shown by the accumulation of lipids and the expression of the adipocytic markers PPARγ, CEBPα and Glut4 Supplementary Figs 1A-C). We co-cultured differentiated adipocytes with AT3 or BTE136 cells, two cell lines derived from MMTV-PyMT tumors, with an epithelial or mesenchymal phenotype, respectively (Supplementary Fig. 1D). We also employed the widely used MCF-7 and SKBR3 cell lines derived from human breast adenocarcinomas (Supplementary Figs 1D-E).

**Figure 1:**
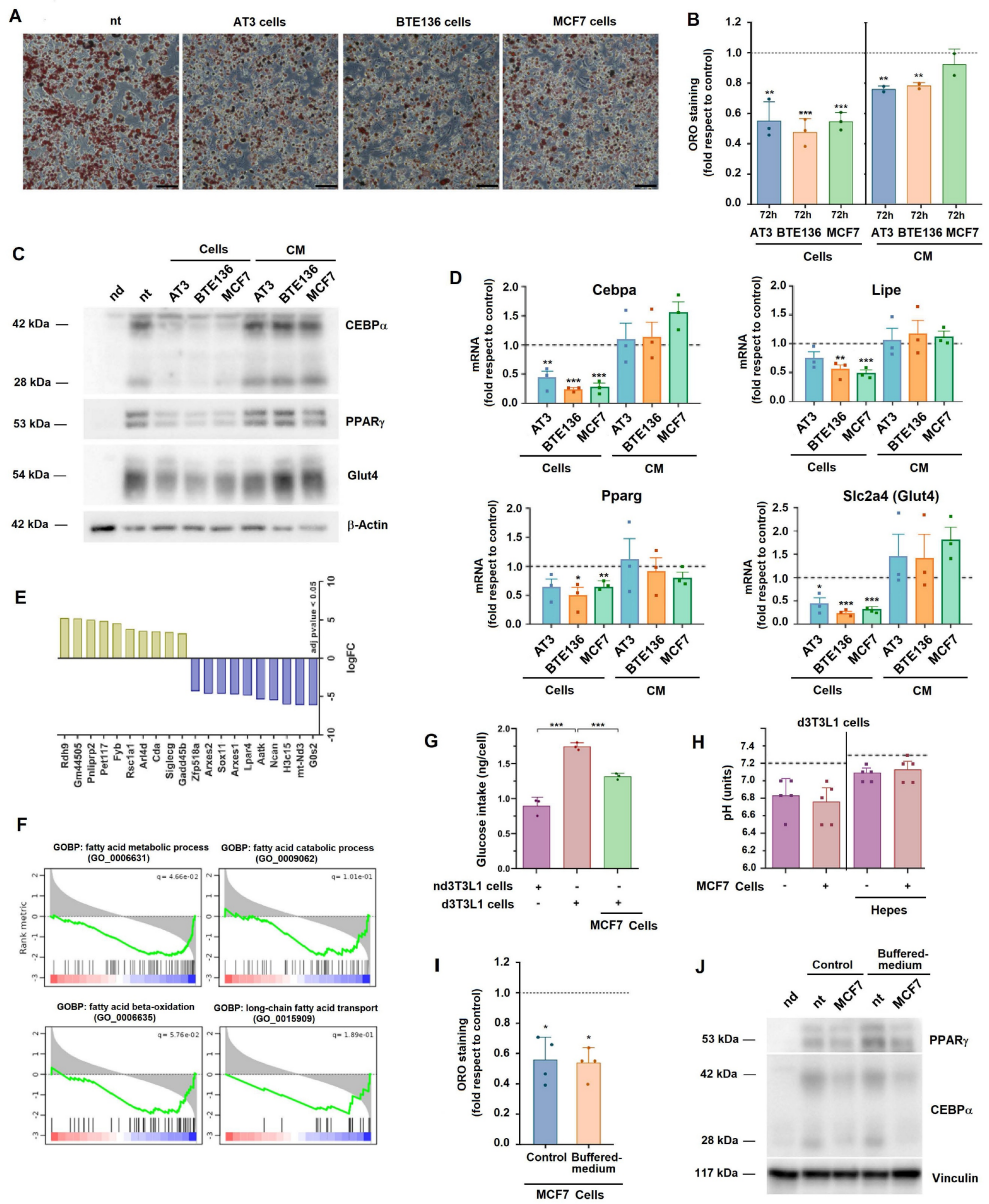
** 3T3L1 adipocytes dedifferentiate when co-cultured with tumor cells.** 3T3-L1 adipocytes were co-cultured with AT3, BTE136, or MCF7 cells for three days or with conditioned medium (CM) derived from these cells. Lipid loss was assessed by ORO staining (**A**) and quantified using ImageJ (**B**); expression of adipocytic markers, by western blot (**C**) or RT-PCR (**D**). In A, scale bar represents 50 μm. In B and D, the control corresponds to non-co-cultured adipocytes. In **E**, bulk RNA was collected from differentiated 3T3L1 adipocytes co-cultured with MCF7 cells when indicated, sequenced and analyzed. The most significantly modified genes in cocultured versus control adipocytes are shown based on an adjusted p-value < 0.05. **F**, GSEA analysis of GOBPs related to fatty acid metabolism. **G**, glucose consumption was assessed as indicated in Methods from not-differentiated (nd) or differentiated (d) 3T3L1 adipocytes cultured with MCF7 when indicated. **H**, the pH of the adipocyte extracellular medium was determined before or after co-culture with tumor cells, in regular DMEM medium or in medium supplemented with 25 mM Hepes pH 7.4. The line indicates the initial pH of cells in both conditions. Lipid loss (**I**) or expression of adipocytic markers (**J**) were quantified as above from adipocytes incubated with MCF7 in regular of Hepes-supplemented medium. In B, D, G, H and I, the average ± SEM of at least three independent experiments is shown. *, p < 0.05; **, p < 0.01; ***, p < 0.001.

We mimicked breast tumors culturing tumor cells in the upper compartment of Transwells and differentiated adipocytes, in the lower one. As shown in Figs 1A and B, under these conditions, adipocytes decreased their lipid content upon incubation with the three cell lines. This loss of neutral lipids was detected after three days and was slightly increased at longer times of co-culture (Supplementary Fig. 2A). It was only partially inhibited by Atglistatin (ATGL), an inhibitor of triglyceride lipase that prevents fatty acid (FA) mobilization from triglycerides (19 Supplementary Fig. 2B). This result suggests that triglycerides loss is partially dependent on its hydrolysis to FA.

Lipid loss correlated with a downregulation of the adipocytic differentiation markers CEBPα, PPARγ, Glut4 (Slc2a4) and Lipe, as confirmed by western blot and RT-PCR (Figs 1C and D). Interestingly, supplementation of differentiated adipocytes with conditioned medium (CM) from tumor cells only partially decreased lipid loss (Fig. 1B) and did not promote significant changes in the expression of the differentiation markers (Figs 1C and D). This indicates that a cross-talk between tumor cells and adipocytes is relevant for their dedifferentiation.

We carried out an extensive mRNA analysis to compare the gene expression pattern of adipocytes stimulated with MCF7 compared to control. A list of the genes showing the highest differences is shown in Fig. 1E. The complete list was deposited in GEO repository (see Methods). The increase in several of these genes was also verified by individual RT-PCR (Supplementary Fig. 2C). Gene set enrichment analysis revealed that biological processes related to fatty acid (FA) metabolism were downregulated in MCF7-treated adipocytes (Fig. 1F). An *in silico* analysis using markers of tumor microenvironmental cells revealed that MCF7 promoted the downregulation of the expression of genes characteristic of mature adipocytes and of master regulators of adipogenic differentiation, as well as up-regulated the expression of those specific of fibroblasts, mesenchymal stem cells or even macrophages (Supplementary Fig. 2D). Another characteristic of adipocytic versus not differentiated 3T3-L1 cells is an increased glucose consumption. As shown in Fig. 1G, MCF7-treated 3T3L1 cells displayed a lower glucose utilization.

Adipocyte lipolysis can be produced by extracellular medium acidification (12. Actually, the pH of the adipocyte culture medium decreased from 7.2 to 6.8 after three days of culture; not significant differences were observed between 3T3L1 cells cultured in the control medium or with tumor cells (Fig. 1H). We controlled acidification adding to the medium a buffering agent. In this condition, the pH remained at 7,1 after three days, either in the absence or presence of tumor cells (Fig. 1H). Even in these conditions, loss of lipids and adipocytic markers was promoted by incubation with tumor cells (Fig. 1I and J).

Several pathways were stimulated in MCF7-treated adipocytes; among them, interleukin and Stat-activated signaling Supplementary Fig. 2E). Accordingly, Stat3 phosphorylation was induced in 3T3-L1 cells by incubation with MCF7 or other tumor cells (Supplementary Fig. 2F). However, incubation of adipocytes with cytokines capable to activate this pathway, such as IL-6 or LIF did not promote lipid loss (Supplementary Figs 2G and H).

### Adipocytes increase tumor cell migration

**Figure 2:**
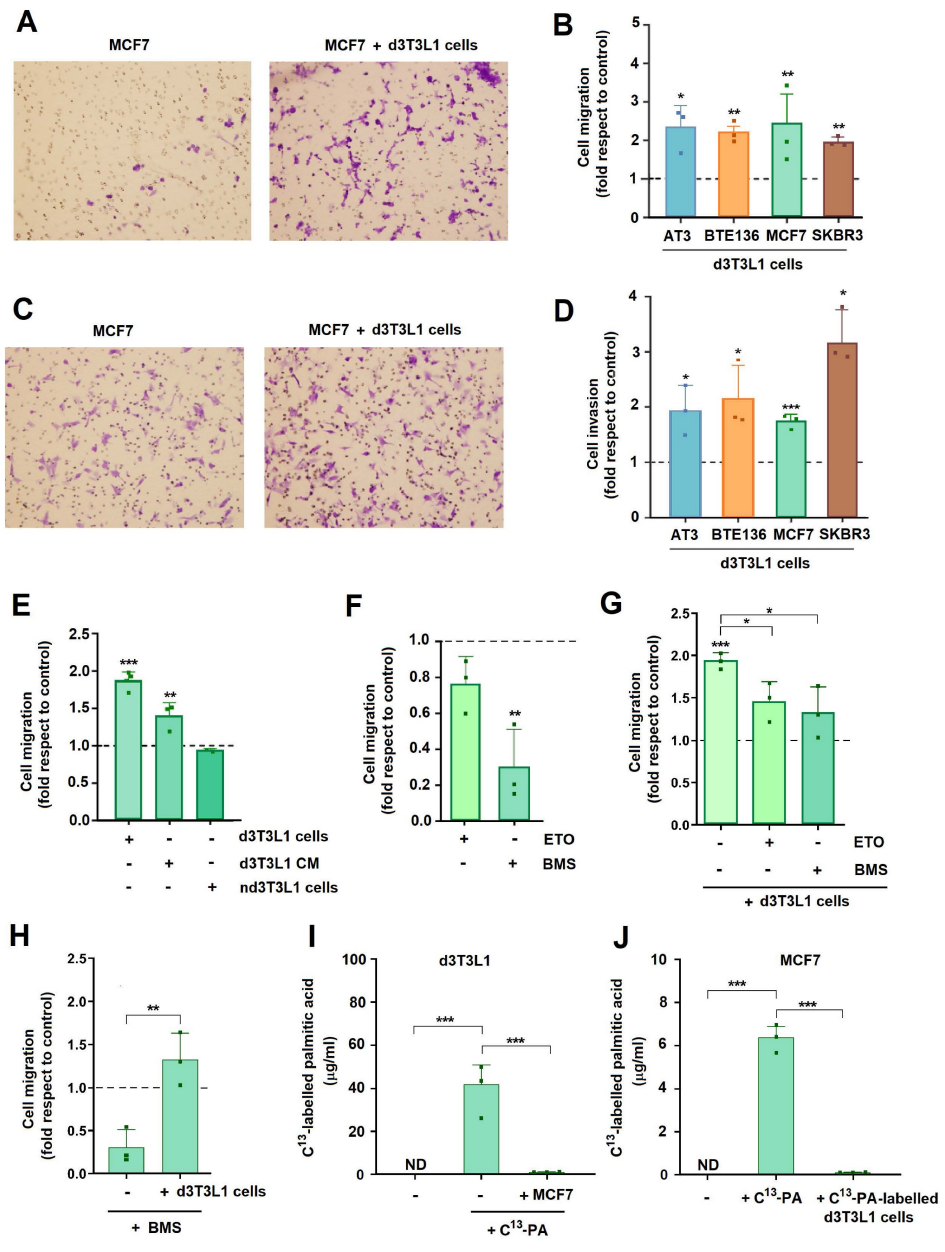
** Adipocytes enhance tumor cell invasion and migration.** Representative images of migration (**A**) or invasion (**C**) of tumors cells in Transwells; when indicated, differentiated 3T3L1 (d3T3L1) were present in the lower chamber. The quantification of different experiments is shown in **B** and **D**. Other migration experiments are presented in **E-H**. Controls represent the migration or invasion of tumor cells in the absence of adipocytes. Etomoxir (ETO) or BMS 309403 (BMS) were added at 30 or 20 μM, respectively. **I**, adipocytes were labelled for three days with ^13^C-palmitate (PA) when indicated; then, cells were incubated in control conditions or with MCF7 for three additional days and remaining ^13^C-palmitate was determined. **J**, MCF7 cells were incubated with ^13^C-palmitate -labelled adipocytes or directly with this fatty acid when indicated. ^13^C-palmitate was determined in these cells after three days of co-culture. The average ± SEM of at least three independent experiments is shown. ND, not detectable; *, p < 0.05; **, p < 0.01; ***, p < 0.001.

Co-culture with differentiated adipocytes increased the migration of MCF7, SKBR3, AT3 and BTE136 tumor cells compared with unchallenged cells, or with cells cultured with not differentiated 3T3L1 cells (Figs 2A and B). Similar results were obtained when cell invasion was determined (Figs 2C and D). This stimulation was observed when migration or invasion were driven by an FBS gradient (from 1 to 10% in the upper and lower chambers, respectively). Addition of CM from differentiated adipocytes also partially mimicked the effect of co-cultured cells on MCF7 migration (Fig. 2E). Very little effect was observed on cell proliferation (Supplementary Fig. 3A).

We reasoned that this migration enhancement might be dependent on FA transfer from adipocytes to tumor cells. To analyze this hypothesis, we used BMS-309403 (BMS), a FA binding protein (FABP) inhibitor that prevented loading of this protein with FAs and, therefore, FA cell uptake (20. Addition of this compound remarkedly decreased MCF7 migration, both basal and adipocyte-induced, suggesting that migration is dependent on MCF7 FA uptake from culture medium (Figs 2F and G). Interestingly, even in the presence of BMS, incubation with adipocytes increased MCF7 migration with respect to the unchallenged cells (Fig. 2H). The inhibitory effect of the FABP inhibitor was prevented by supplementation of the medium with FAs, to compete with the binding of the inhibitor to FABP Supplementary Fig. 3B). The relevance of FA in MCF7 migration was also highlighted by experiments using another inhibitor of FA metabolism such as Etomoxir (ETO) that inhibits FA oxidation (21. This compound also decreased both basal and adipocyte-induced migration in FBS gradients (Figs 2F and G). No significant effects on MCF7 proliferation were observed with the concentrations of BMS and ETO used in these assays Supplementary Fig. 3C).

Altogether, these results suggest that FA taken from adipocytes or from the regular culture medium are incorporated by MCF7 and further metabolized through FA oxidation.

We analyzed the FAs content from the CM from 3T3L1 adipocytes or MCF7 cells. As shown in Supplementary Fig. 3D, CM from MCF7 cells co-cultured with 3T3L1 adipocytes do not show increased levels of most predominant FAs (myristic, palmitic or stearic acids; C14:0, C16:0 or C:18:0, respectively) when compared with CM from not treated MCF7 cells. Moreover, CM from 3T3L1 adipocytes only present a slight increase in these FAs with respect to the culture medium (Supplementary Fig. 3E). Only some low abundant FAs (pentadecanoic, C15:0; heptadecanoic, C:17:0; or palmitoleic, C16:1n7) were increased in these conditions. This suggests that adipocytes-derived lipids do not contribute significantly to the total amount of FAs in the CM.

Differentiated 3T3L1 cells were incubated with ^13^C-labelled palmitic acid that was incorporated into the adipocytes (Fig. 2I). Following incubation with tumor cells, adipocytes showed an almost complete loss in ^13^C-labeled palmitic acid (Fig. 2I). MCF7 cells also incorporate palmitic acid when this compound was supplemented to the CM although to a lower extent than adipocytes (Fig. 2J). Although detectable, FA transfer from labelled adipocytes to MCF7 was very low (Fig. 2J), suggesting that these cells uptake palmitic acid directly from the CM.

Finally, addition of myristate, palmitate or other FAs did not increase MCF7 migration although they were incorporated by these cells (Supplementary Figs 3F and G).

These results indicate that in our cellular system FAs are taken mostly from the cell medium and are required for migration but are not capable to promote this process by themselves.

### Tumor cells undergo a partial epithelial-to-mesenchymal transition (EMT) upon co-culture with adipocytes

**Figure 3:**
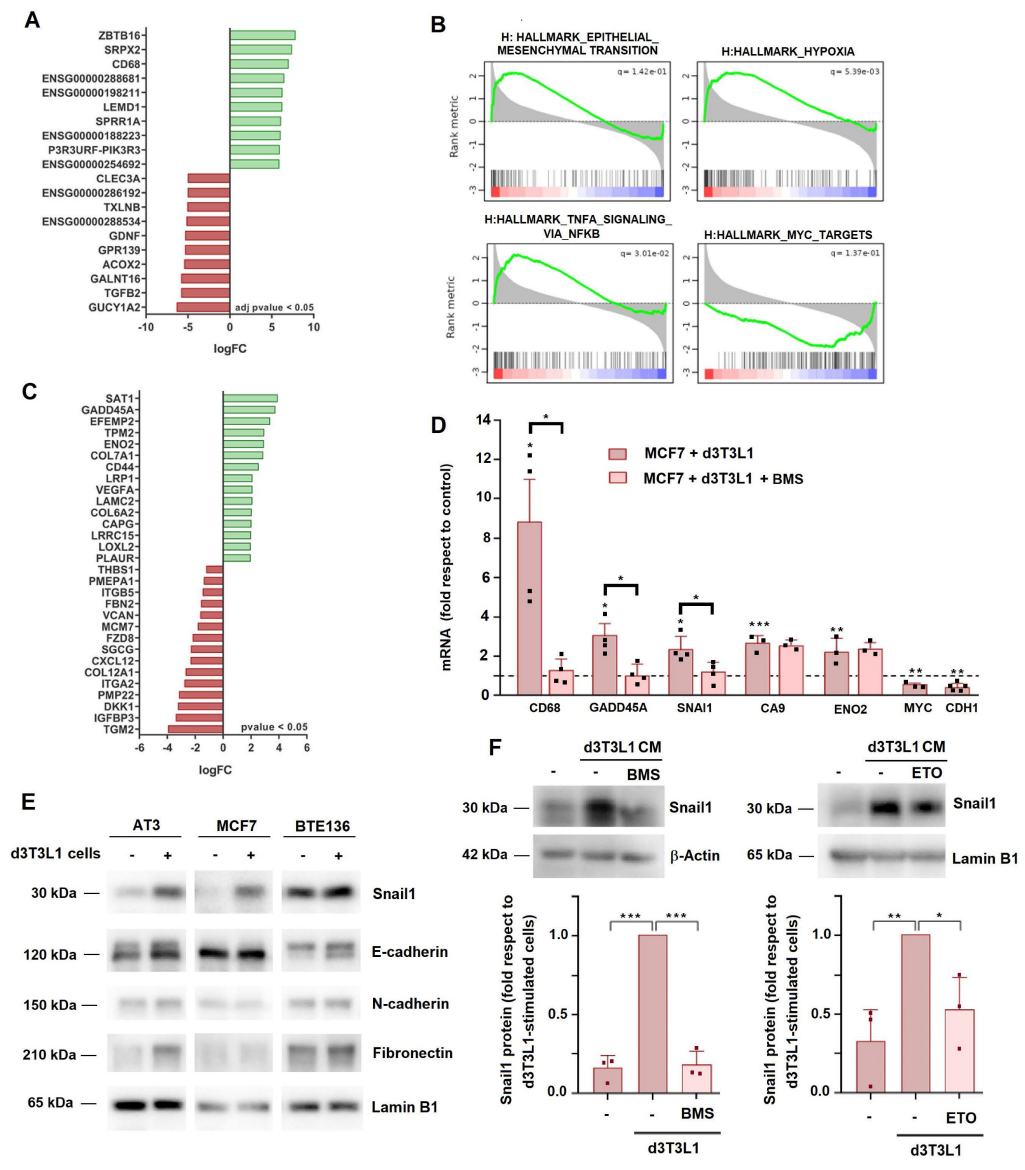
** Adipocyte co-culture promotes a partial EMT in tumor cells.** MCF7 cells were co-cultured with d3T3L1 adipocytes for three days. Total RNA was collected, mRNA sequenced and analyzed. The most significantly modified genes in adipocyte-cocultured versus control MCF are shown in **A**, GSEA of the top modified hallmarks in **B**, and the genes related to EMT and hypoxia exhibiting the highest differences, in **C**. **D-F**, MCF7, AT3 or BTE136 cells were incubated with differentiated 3T3L1 adipocytes (or with CM from these cells) for three days and levels of the indicated proteins (E, F) or RNAs (D) were assessed. In D, the control (dashed line) corresponds to untreated MCF7 cells. In F, lower panel, three different western blots as those shown in the upper panel were quantified as described in Supplementary Methods. Values were represented relative to the Snail1 level in MCF7 cells stimulated with CM from differentiated 3T3L1 cells. ETO and or BMS were added at 30 μM and 20 μM, respectively. The average ± SEM of at least three independent experiments is shown. *, p < 0.05; **, p < 0.01; ***, p < 0.001.

We carried out an extensive RNA analysis to compare the gene expression pattern in tumor cells upon two days of co-culture with adipocytes. A list of the RNAs showing the highest expression differences is shown in Fig. 3A; the complete list was deposited in GEO repository (see Methods). Gene set enrichment analysis (GSEA) revealed that co-culture with adipocytes modified the expression in MCF7 of genes related to EMT or other pathways related to this conversion, such as hypoxia and TNFα signaling (Fig. 3B). A list of the genes related to EMT is presented in Fig. 3C. The modulation of several of these genes (CD68, GADD45A, CA9, ENO2) was also validated by RT-PCR (Fig. 3D). We also analyzed the expression of other proteins commonly associated to EMT: only Snail1 and fibronectin were consistently stimulated in these conditions; small differences were detected in mesenchymal N-cadherin (CDH2) or epithelial E-cadherin (CDH1) (Figs 3D and E). In contrast to other cellular models in which Snail1 is rapidly activated upon addition of the stimulus triggering the EMT, Snail1 upregulation was slow and required three days of co-culture (Supplementary Fig. 4A). Snail1 activation was also detected in tumor cells when they were co-cultured with primary adipocytes (Supplementary Fig. 4B). Adipocyte CM increased the levels of SNAI1, CD68 and GADD45A mRNAs in MCF7 cells (Supplementary Fig. 4C) and also of Snail1 protein, particularly in MCF7 and AT3 cells that express lower endogenous levels of this protein (Fig. 3E and Supplementary Fig. 4D).

Interestingly, similar to cell migration, the increase expression of the mesenchymal markers was sensitive to BMS; this compound inhibits SNAI1, CD68 and GADD45A RNA up-regulation by adipocytes (Fig. 3D) and the Snail1 protein increase promoted by CM from these cells (Fig. 3F). ETO also prevented Snail1 stimulation by adipocytes (Fig. 3F and Supplementary Fig. 4E). However, the effect of BMS on adipocyte-stimulated gene expression was not general and some genes were not affected by this compound (Fig. 3D).

Taken together, these results suggest that tumor cells FA metabolism is required for the induction by adipocytes of mesenchymal markers such as Snail1 but other elements also participate in this stimulation.

### Adipocytes promote ROS production in tumor cells

**Figure 4:**
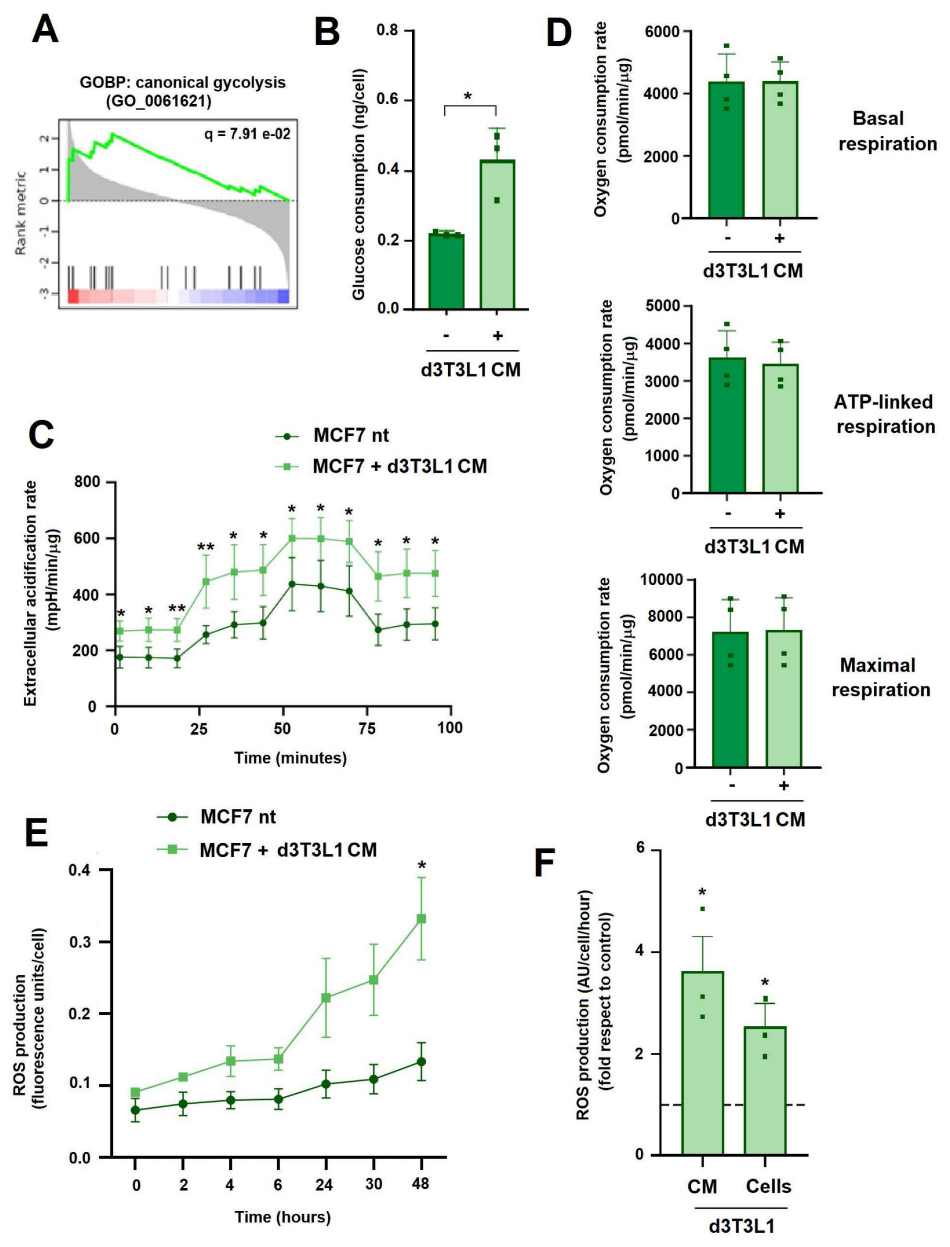
** Adipocytes increase ROS production in tumor cells. A,** GSEA of the top modified GOBPs with an FDR < 0.2.** B,** glucose concentration was determined in the cellular medium of MCF7 cells co-cultured with d3T3L1 CM for three days. **C-D**, Seahorse determinations of extracellular acidification rate, basal respiration, ATP-linked respiration or maximal respiration in MCF7 cells treated for three days with d3T3L1 CM. **E**, kinetics of ROS generation in MCF7 treated with d3T3L1 CM. **F**, ROS production was determined in MCF7 after two days of culture with d3T3L1 CM or with these cells. The average ± SEM of three independent experiments is shown. *, p < 0.05; **, p < 0.01.

The analysis of genes activated in MCF7 by coculture with adipocytes revealed that biological processes related to glycolysis were activated in these cells (Fig. 4A). Therefore, we analyzed the changes in MCF7 glucose metabolism. Upon incubation with adipocytes or adipocyte-conditioned medium MCF-7 cells consumed more glucose (Fig. 4B) and presented a higher extracellular acidification rate (ECAR) (Fig. 4C), indicative of an increased glycolysis. In contrast, d3T3L1 CM did not modify several parameters related to mitochondrial activity such as basal, ATP-linked or maximal respiration (Fig. 4D). Remarkably, MCF7 treated with adipocytes CM generated more reactive-oxygen species (ROS) (Figs 4E and F), a rise that was more evident after 6 hours of incubation (Fig. 4E). This ROS increase was not inhibited by ETO or BMS (Supplementary Fig. 5A), further suggesting that is not caused by mitochondrial FA oxidation. Therefore, the increase in tumor cell migration caused by adipocytes was accompanied with an elevated glucose utilization and cytoplasmic ROS generation.

### ROS generation is required for tumor migration and invasion

**Figure 5:**
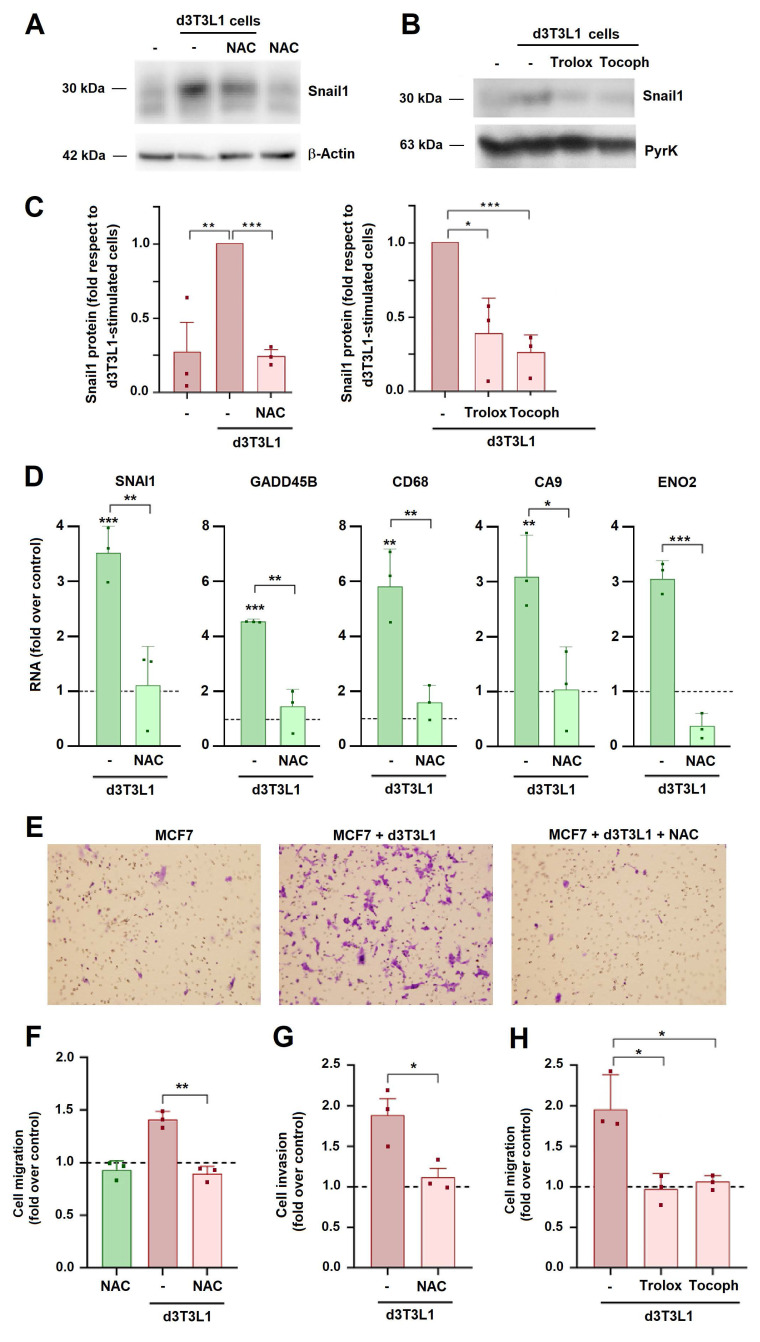
** N-acetyl-cysteine (NAC) prevents adipocyte effects on MCF7.** MCF7 cells were co-cultured with d3T3L1 for three days in the presence of NAC (1 mM), Trolox (10 μM), or Tocopherol (10 μM) and Snail1 protein (**A-C**) or the indicated RNAs (**D**) were analyzed. In C, the quantification of three different western blots is presented; the control corresponds to the value obtained in MCF7 cells stimulated with 3T3L1 adipocytes. Migration (**E, F, H**) or invasion (**G**) of MCF7 cells was assessed after three days of co-culture with adipocytes and with the indicated treatments. The control corresponds to MCF7 cultured in DMEM medium. Representative images of a migration experiment are shown in E. The average ± SEM of at least three independent experiments is shown. *, p < 0.05; **, p < 0.01; ***, p < 0.001.

We assessed the role of generated ROS in tumor cell activation. For this, we used N-acetyl-cysteine (NAC), a well-known ROS scavenger. As expected, this compound decreased ROS in MCF7 cells (Supplementary Fig. 5B). NAC, as well as two other ROS quenchers, Tocopherol and Trolox (Supplementary Fig. 5C), significantly inhibited adipocyte-induced Snail1 expression in MCF7 cells (Figs 5A-C). The stimulation of SNAI1 or other genes stimulated by adipocytes was also affected by NAC (Fig. 5D). NAC, Trolox and Tocopherol also decreased 3T3L1-induced MCF7 invasion and migration (Figs 5E-H).

**Figure 6:**
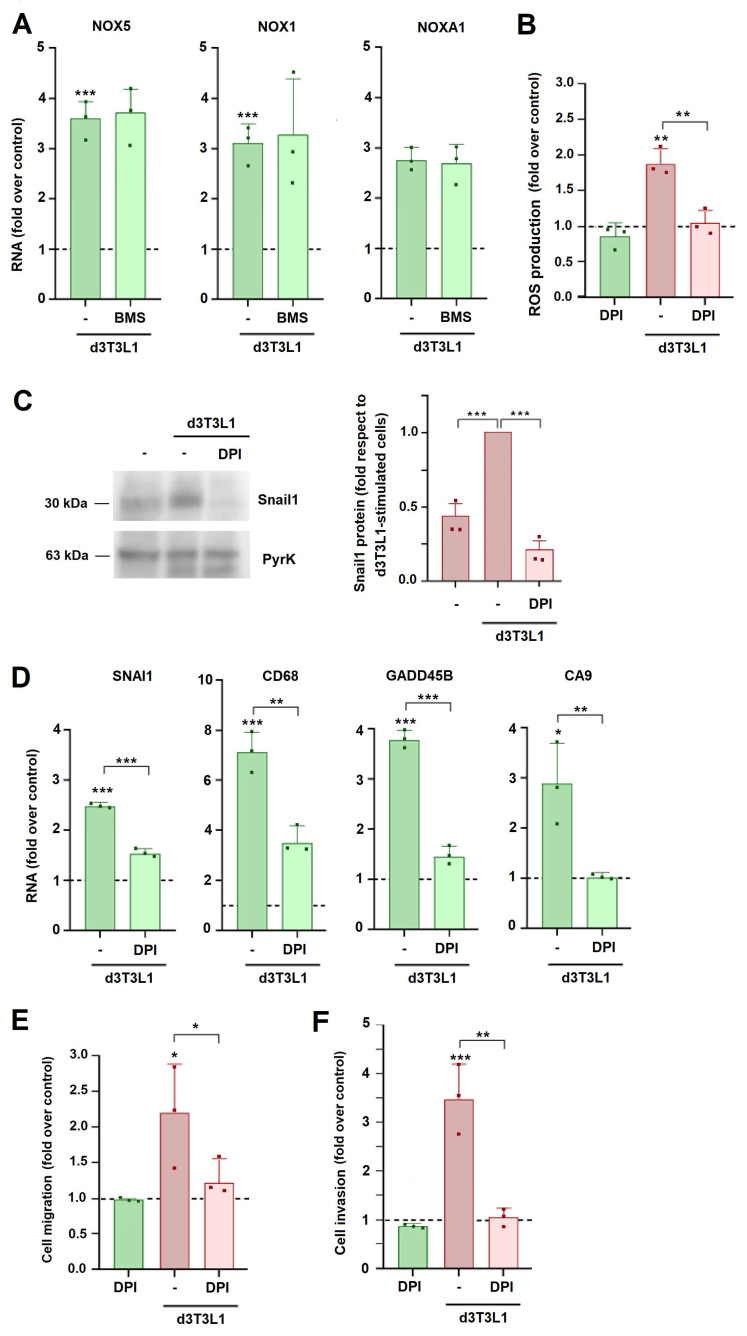
** NOX activity is required for adipocyte-induced MCF7 partial EMT and migration.** MCF7 cells were cultured with d3T3L1 in the presence of BMS (20 μM) or DPI (0.5 μM) when indicated; RNA expression (**A, D**), ROS (**B**), Snail1 protein (**C**), migration (**E**) and invasion (**F**) were assessed. In C, the right panel corresponds to the quantification of three different western blots. The control corresponds to MCF7 cultured in DMEM medium. The average ± SEM of at least three independent experiments is shown. *, p < 0.05; **, p < 0.01; ***, p < 0.001.

We considered that the increase in ROS might be consequence of an elevation in the expression of NADH oxidases (NOX). Among the four NOX proteins, only NOX1 and NOX5 were detected in MCF7 cells; expression of these genes was up-regulated by co-culture with adipocytes (Fig. 6A). NOX1 activator NOXA1 was also increased in these conditions. The stimulation in the expression of these mRNAs was not affected by BMS (Fig. 6A), indicating that was independent on FA metabolism, in accordance with the results obtained when ROS levels were determined (see Supplementary Fig. 5A).

Diphenyleneiodonium (DPI), a well characterized NOX inhibitor (22, decreased ROS generation and Snail1 expression induced by adipocyte co-culture (Figs 6B and C). Expression of other genes associated to the EMT and stimulated by adipocytes was also affected the inhibitor (Fig. 6D). DPI also prevented the increase in MCF7 migration and invasion induced by co-culture with 3T3L1 adipocytes (Figs 6E and F).

**Figure 7:**
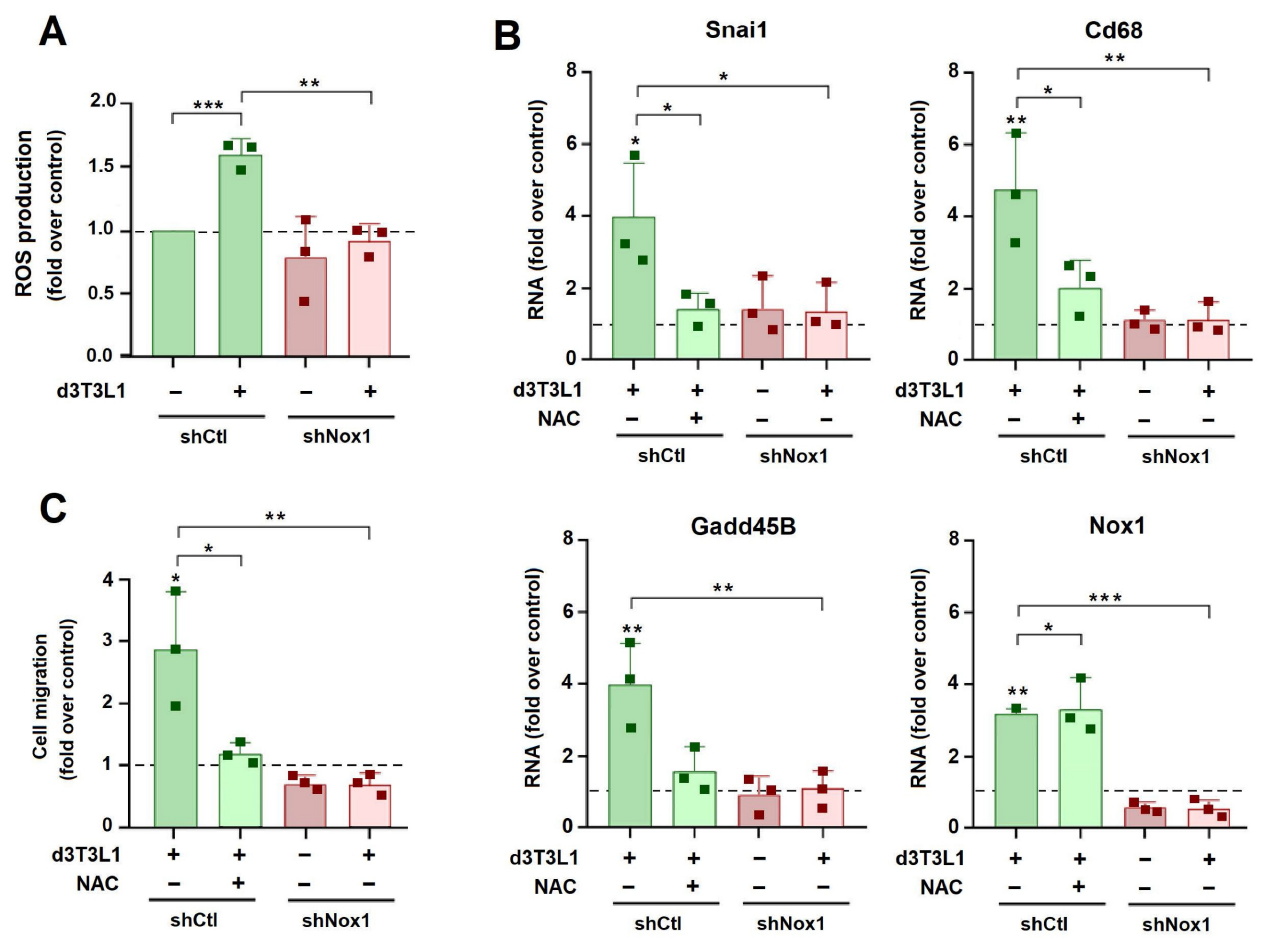
** Nox1 is required for adipocyte-induced EMT and migration of AT3 cells.** AT3 cells, either transfected with shCtl or shNox1 (sh1.4), were cultured with d3T3L1 and NAC (1 mM) when indicated, and ROS (**A**), the indicated RNAs (**B**) or cell migration (**C**) were determined. The control corresponds to AT3 cells transfected with shCtl and cultured in DMEM medium. The average ± SEM of at least three independent experiments is shown. *, p < 0.05; **, p < 0.01; ***, p < 0.001.

We also genetically depleted NOX in tumor cells. For these experiments we used murine AT3 cells since the murine genome does not contain the NOX5 gene 23. Similar to MCF7 cells, co-culture with adipocytes increased the expression of Snail1 and other mesenchymal genes in AT3 cells. This induction was prevented by NAC Supplementary Fig. 6A). Nox1 was also stimulated but was not sensitive to NAC. We reduced Nox1 expression in AT3 using a specific shRNA (Supplementary Fig. 6B). This Nox1 drop blunted the ROS increase induced by co-incubation of AT3 with adipocytes (Fig. 7A). Expression of Snail1 and other mesenchymal markers was also decreased to a similar extent by shNox1 than by NAC addition (Fig. 7B). AT3 migration was stimulated by 3T3L1 adipocytes; this increase was prevented by addition of NAC or by Nox1 down-regulation (Fig. 7C).

## Discussion

This article investigates how a TME cellular component, adipocytes, promotes tumor cell migration and invasion. Our experimental model, in which adipocytes and tumor cells were cultured in different chambers of a Transwell, reveals that both cell types cross-talk. First, adipocytes dedifferentiate when co-cultured with tumor cells, as demonstrated by the loss of lipids and down-regulation of adipocytic markers. Although the loss of these markers is not total, these dedifferentiated adipocytes start to express genes specific of fibroblasts, mesenchymal stem cells or even macrophage precursors (see Supplementary Fig. 2E). A similar transformation of adipocytes in fibroblast and macrophage-like cells has been observed by other authors (14. Adipocyte dedifferentiation by tumors cells was more extensive when both cells were co-cultured than when adipocytes were treated with conditioned medium of tumor cells. This could be explained by the possibility that MCF7 cells need prior activation by adipocytes in order to secrete factors that promote dedifferentiation. An alternative explanation, although less likely, is that the process is initiated by tumor cells that have migrated into the lower, adipocyte-containing compartment.

Adipocyte loss of neutral lipids was partially but not totally inhibited by Atglistatin, an inhibitor of triglyceride lipase, that prevents FA mobilization from triglycerides 19. This result suggested us that, although adipocytes metabolize triglycerides to FA and secrete these molecules, they might be also losing triglycerides directly, maybe through the secretion of vesicles containing lipid droplets. Accordingly, adipocytes-derived vesicles have been reported to facilitate FA oxidation in tumor cells 24.

Besides undergo this process of dedifferentiation, adipocytes enhance tumor cell migration and invasion. Migration was sensitive to inhibitors of FA transport (BMS) and catabolism (ETO) suggesting that tumor cells require FAs to migrate. Different reports have demonstrated that metastatic tumor cells are particularly addicted to FA metabolism 8, 25, 26. Actually, BMS significantly affects growth of tumor cells *in vivo* 27, 28. However, even in the presence of BMS, adipocytes enhance tumor migration (see Fig. 2). This suggested us that, although FAs are required, an additional adipocyte-derived factor is the main responsible for the enhanced tumor migration. Two other results support this conclusion. First, addition of different FAs did not promote tumor cells migration. Second, in our cellular model tumor cells take these compounds mostly from extracellular medium. Actually, when we assessed the total amount of FAs in the MCF7 cell culture medium, presence of differentiated adipocytes only slightly increased the concentration of the most abundant FAs, such as myristic or palmitic acids. Experiments of FA transfer from adipocytes to tumor cells also showed that this transfer is low. Therefore, we conclude that the main effect of adipocytes in our cellular model consists in promoting a partial EMT and migration. Although FA metabolism by tumor cells is required for this process, these compounds are mostly taken from the CM.

This adipocyte-induced partial EMT of tumor cells is characterized by a slow increase in Snail1 and other mesenchymal markers. Similar to migration, this increase in the expression of Snail1 and other genes requires FAs since it is partially inhibited by BMS and ETO. It remains to be investigated how FA oxidation impacts on Snail1 expression.

Moreover, adipocytes elevated tumor cells glucose utilization and glycolysis but did not alter their mitochondrial activity, in accordance with previous results 29. We also observed a higher production of ROS, that is not a consequence of FA metabolism since it is not inhibited by BMS or ETO. Alternatively, ROS increase is produced by the action NOX proteins 1 and 5 that are up-regulated by co-culture with adipocytes. A similar rise of NOX5 in tumor cells has been reported by other authors 30. However, we cannot discard that in other cellular systems other NOX proteins (i.e. NOX4) might also play a role. NOX are required for ROS generation and for the increase in Snail1 and other mesenchymal markers. Accordingly, a NOX inhibitor prevents the up-regulation of EMT markers in tumor cells stimulated by adipocytes and the increase in tumor migration and invasion. Similar effects were observed genetically depleting NOX1 in murine cells, that lack NOX5. However, it is not known yet how NOX expression is up-regulated by adipocytes and if this involves specific cytokines. In any case, it is not sensitive to inhibitors of FA metabolism. Therefore, Snail1 expression, cell migration and invasion are dependent on two specific pathways: one dependent on FA metabolism and another driven by NOX-dependent ROS generation.

In accordance to these results, inhibitors of FA metabolism and NOX are being actively pursued for clinical use. Regarding FAs, many different inhibitors of their uptake and metabolism have been characterized 31. Although most are in preclinical phases, some as denifastat are in clinical trials 32. The NOX4 inhibitor setanaxib (GKT137831) is the first compound to advance to cancer clinical trials for the treatment of metastatic squamous carcinoma of head and neck 33. Moreover, the effect of this compound on the activation of cancer-associated fibroblasts 34, 35, a process with many mechanistic similarities to EMT, suggests that this compound might be useful in many other neoplasms.

Our study presents several limitations. For instance, our hypothesis should be verified in *in vivo* tumorigenesis experiments. Studies performed generating mammary gland tumors in NOX1-depleted animals should be very informative. Moreover, some mechanistic aspects need to be better investigated. It is not known how ROS regulates the expression of Snail1 and other mesenchymal markers. It has been proposed that ROS might activate HIF1 expression and activity 29, and HIF signaling has already been associated to EMT 36. Indeed, in our cellular model expression of genes stimulated by hypoxia is increased in tumor cells by incubation with adipocytes (see Fig. 3b). However, Snail1 up-regulation in hypoxia is mostly post-translational 37, 38, whereas a substantial increase in Snail1 RNA is detected in our conditions. Alternatively, or additionally, Snail1 activation by ROS has been reported to be mediated by NF-κB 39, a factor that stimulates Snail1 transcription and protein stability 40, 41. ROS can also promote the activation of Akt 42, a protein kinase that stimulates the expression of Snail1 and other mesenchymal markers 43. These pathways might be inter-related since NF-kB can be activated by Akt 44. In any case, further research is needed to elucidate the role of ROS in EMT in this and other cellular models and characterize its downstream effectors required for EMT and tumor invasion.

## Supplementary Material

Supplementary materials and methods, figures and tables.

## Data Availability

All the data generated during the current study are available from the corresponding author on reasonable request.

## Abbreviations

ATGL: triglyceride lipase inhibitor Atglistatin
BMS: fatty acid binding protein inhibitor BMS-309403
CAA: cancer-associated adipocyte
CEBPα: CCAAT/enhancer-binding protein alpha
CM: conditioned medium
d3T3L1: differentiated 3T3L1 cells
DMEM: Dulbecco's modified Eagle's medium
DPI: NOX inhibitor diphenyleneiodonium
EMT: epithelial-to-mesenchymal transition
ETO: fatty acid oxidation inhibitor Etomoxir
FA: fatty acid
FABP: fatty acid binding protein
FBS: fetal bovine serum
NAC: N-acetyl cysteine
PBS: phosphate-buffered saline solution
NOX: NADPH oxidase
ORO: Oil Red O
PPARɣ: peroxisome proliferator-activated receptor
ROS: reactive oxygen species
SDS: sodium dodecyl sulphate
SRM: Selected Reaction Monitoring
TME: tumor microenvironment
