# Supplementary material for: A crosstalk between tumor cells and adipocytes facilitates tumor cell migration and invasion
Source: Int J Biol Sci. 2026 Mar 4;22(6):3272–87. doi: 10.7150/ijbs.117604 (PMC13050473; doi:10.7150/ijbs.117604)
Supplement: Supplementary file 1 — Supplementary materials and methods, figures and tables. [file ijbsv22p3272s1.pdf]

A crosstalk between tumor cells and adipocytes facilitates tumor cell migration and invasion

Laura Garrido-Jiménez, Beatriz del Valle-Pérez, Javier Pastor, Élida Alechaga, Aleix Sala-Vila, Óscar J. Pozo, Raúl Peña, and Antonio García de Herreros

Supplementary Materials and Methods

Fatty acid analysis. Cell supernatants were collected and stored at -80°C until fatty acid analysis, which was performed at Hospital del Mar Research Institute (Barcelona, Spain). Samples were spiked with the internal standard (ISTD) glyceryl trinonadecanoate (Avanti, Merck) into a chloroform-resistant Eppendorf. Lipids were extracted with chloroform–methanol (2:1, v/v) containing butylated hydroxytoluene (50 mg/ml) and evaporated to dryness under N2 at 37ºC. The lipid extract was re-dissolved in toluene and transferred to a screw-cap test-tube. Fatty acid methyl esters (FAMEs) were prepared by incubation with acidified methanol, as described (1). FAMEs were analyzed by gas chromatography/electron ionization mass spectrometry (GC-MS), using an Agilent 6890N GC equipped with an Agilent 7683 autosampler and an Agilent 5973N mass spectrometry detector. FAMEs were separated with a J&W DB-FastFAME capillary column (30 m × 0.2 mm × 0.25 µm film thickness) (Agilent). Injections of 1 µL were performed (split ratio 25:1). FAMEs were quantified using the selected ion monitoring (SIM) mode. Based on the work of Thurnhofer and Vetter (2), several m/z ions common to saturated, monounsaturated, and polyunsaturated FAMEs were monitored. Twelve mixtures of FAME external calibration standards, spiked with C19:0-methyl ester in an equivalent amount to that included in samples as ISTD, were prepared by diluting FAME mix certified reference material (Supelco 37 Component FAME Mix, Merck) in hexane. The concentrations of 24 different FAMEs in the samples were calculated by linear regression of the peak area ratio relative to that of the ISTD. Fatty acid concentration was expressed as mg/l of supernatant.

RNA isolation and analysis. RNA was obtained using RNeasy Mini Kit (Qiagen, 74106), retrotranscribed and analyzed by quantitative PCR (RT-PCR) in triplicate using *HPRT* as control. The list of the primers used is provided in the Suppl Table 1.

Protein analysis by western blot. Cells were scraped from the plate and lysates were obtained using sodium dodecyl sulfate (SDS) lysis buffer (50 mM Tris pH 7.5, 10% Glycerol, 2% SDS) and electrophoresed on 10% SDS-PAGE gels with TGS buffer (25 mM Tris pH 8.3, 192 mM glycine, 0.1% SDS). Gels were then transferred to polyvinylidene difluoride membranes (Merck Millipore) after activating them with methanol, and washed with transfer buffer 1X (25 mM Tris pH 8.3, 192 mM glycine, 10% methanol). After blocking in TBS-T (TBS 0.1% Tween-20) with 1% BSA, the blots were probed with the specified primary antibodies (See Suppl Table 2) and HRP-conjugated secondary antibodies (goat anti-mouse, Invitrogen, 31430; or goat anti-rabbit, Abcam, ab205718). After three washes with TBS-T after antibodies incubation, the blots were incubated with Immobilion® Western Chemiluminiscent HRP Substrate (Merck Millipore) and visualized using (Uvitec Cambridge Alliance Q9 Chemiluminiscent Imager). When indicated, protein levels were quantified using ImageJ software, normalized to the loading control and represented versus the value of d3T3L1-stimulated MCF7 cells.

Migration and invasion assays. Migration and invasion assays were performed using the Boyden chamber system as described (3). For migration assays, 1 x10^5 tumor cells were resuspended in DMEM plus 0.1% FBS in a final volume of 150 µl, seeded on 8 µm-pore size transwells (3422, Costar) and incubated for 48 h. For invasion assays, transwells were first coated with 50 µl of Matrigel (0.5 µg/µl) (354230, Cultek) and incubated for at least 30 min at 37ºC. In both migration and invasion assays, DMEM 10% FBS was used as chemoattractant in the lower compartment with or without differentiated 3T3L1 cells.

After 48 h, cells were fixed with 100% cold MetOH for 20 min at -20ºC. Non-migrating and non-invading cells were removed from the upper compartment of the transwell with a cotton swab. Migrating and invading cells were stained with Crystal Violet for 15 min at room-temperature. After three washes with PBS, the membrane was cut with a scalpel and cells were rinsed with 30% acetic acid (300 µl). Absorbance was analyzed at 590 nm in an Infinite M200 Microplate Reader (Tecan).

Cell proliferation assays. 2 x10^3 AT3 cells, 1.5 x10^3 BTE136 cells or 2.5 x10^3 MCF7 cells were seed in 96 well plates. After 24 h, media was removed and DMEM (plus 10% FBS) or d3T3L1 CM (10% FBS) was added. After three days, proliferation was assessed by Crystal Violet staining. Media was removed, cells were washed three times with PBS and fixed with 100% cold MetOH for 20 min at -20ºC. After three washes with PBS, cells were incubated with 0,25% Crystal Violet for 15 min at room temperature. Finally, stained cells were washed three times with PBS and rinsed with 30% acetic acid (200 µl). Absorbance was analyzed at 590 nm in an Infinite M200 Microplate Reader.

References:

1. Burdge GC, Wright P, Jones AE, Wootton SA. A method for separation of phosphatidylcholine, triacylglycerol, non-esterified fatty acids and cholesterol esters from plasma by solid-phase extraction. Br J Nutr. 2000, 84: 781-7.

2. Thurnhofer S, Vetter W. A gas chromatography/electron ionization-mass spectrometry-selected ion monitoring method for determining the fatty acid pattern in food after formation of fatty acid methyl esters. J Agric Food Chem. 2005, 53: 8896-903.

3. Alba-Castellón L, Olivera-Salguero R, Mestre-Farrera A, Peña R, Herrera M, Bonilla F, et al. Snail1-Dependent Activation of Cancer-Associated Fibroblast Controls Epithelial Tumor Cell Invasion and Metastasis. Cancer Res. 2016, 76, 6205-17.

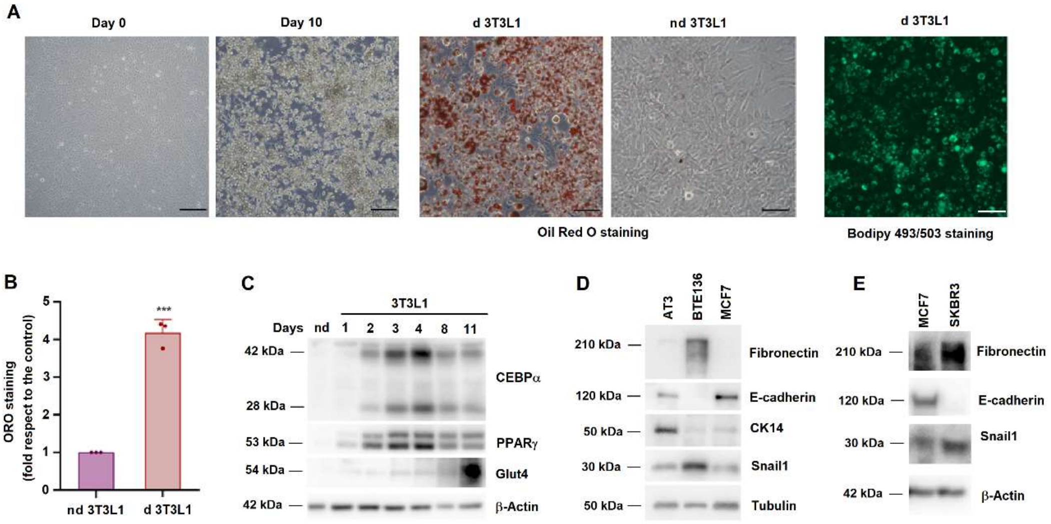

Suppl. Figure 1. **Cellular model used in this work**. The differentiation of 3T3L1 cells was induced when the cells reached confluence as indicated in Methods. Lipid accumulation was assessed in cells at day 12 with ORO and Bodipy 493/503 staining (**A, B**). In **C**, presence of adipocytic markers was analyzed by Western blot. The expression of mesenchymal or epithelial markers in the tumoral cell lines used is shown in **D** and **E**. nd, not differentiated adipocytes; d, differentiated adipocytes. In A, the scale bar corresponds to 50 μm. In B, the average ± SEM of three independent experiments is shown. ***, $p < 0.001$.

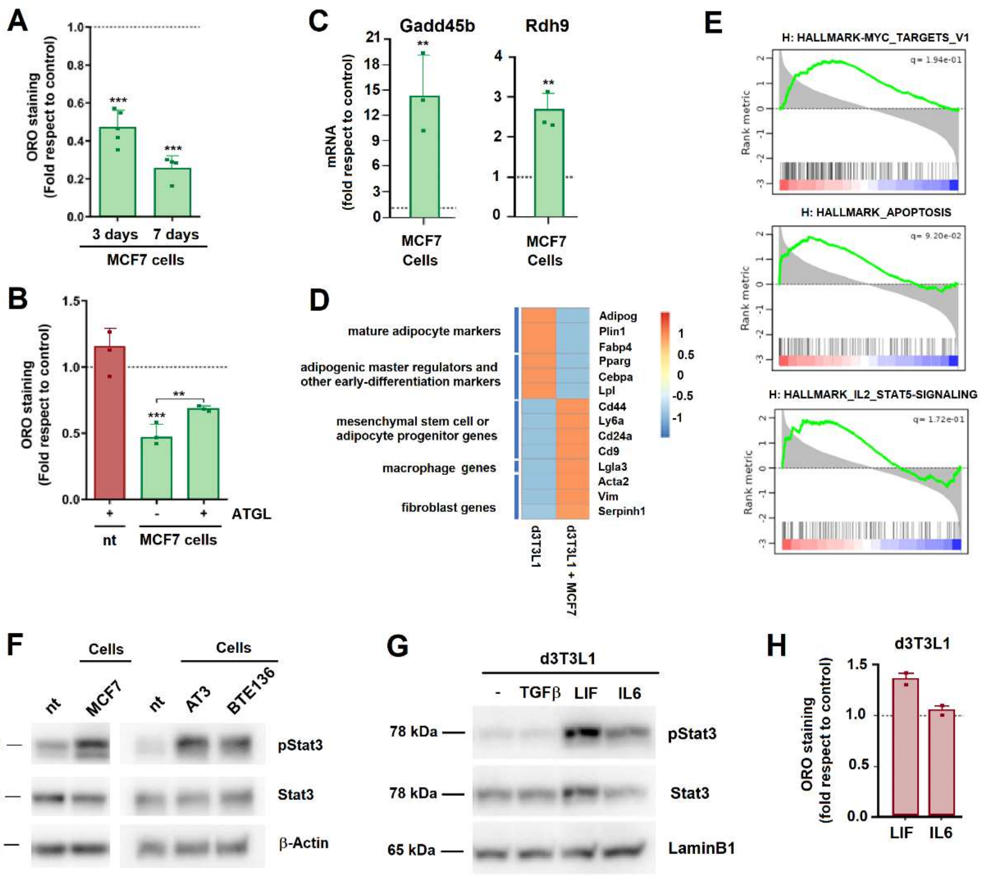

Suppl. Figure 2. **3T3L1 adipocytes de-differentiate in co-culture with tumor cells**. 3T3L1 adipocytes (d3T3L1) were cultured with MCF7 breast tumor cells for 3 (**A-E**) and 7 days (**A**). Lipid loss was assessed by ORO staining and quantified with ImageJ (A-B). Atgilstatin (ATGL, 50 μM) was added when indicated. The dotted line corresponds to the value obtained in untreated adipocytes. In C, total RNA was obtained from adipocytes and analyzed by RT-PCR. The relative expression in MCF7-co-cultured versus control adipocytes (dotted line) is shown. The p value was calculated versus control cells except when ATGL was added in B. D, Heatmap depicting gene expression levels of mature adipocyte markers, early differentiation markers, adipocyte progenitor markers, and macrophage markers, and compared with genes expressed in 3T3-L1 adipocytes either non-co-cultured or directly co-cultured with MCF7 cells for 3 days. E, GSEA analysis of GOBPs related to the top modified hallmarks (H) with an FDR < 0.2. **F**, Stat3 phosphorylation was analyzed by WB in tumor cell-co-cultured

versus control 3T3L1 adipocytes. **G-H**, 3T3L1 adipocytes were treated with TGFβ, IL6 or LIF for 3 days. Stat3 activation was assessed by Western blot (G) and lipid loss by ORO staining (H) as above. The average ± SEM of at least three independent experiments is shown in A-C; the average ± range of two, in H. **, $p < 0.01$; ***, $p < 0.001$.

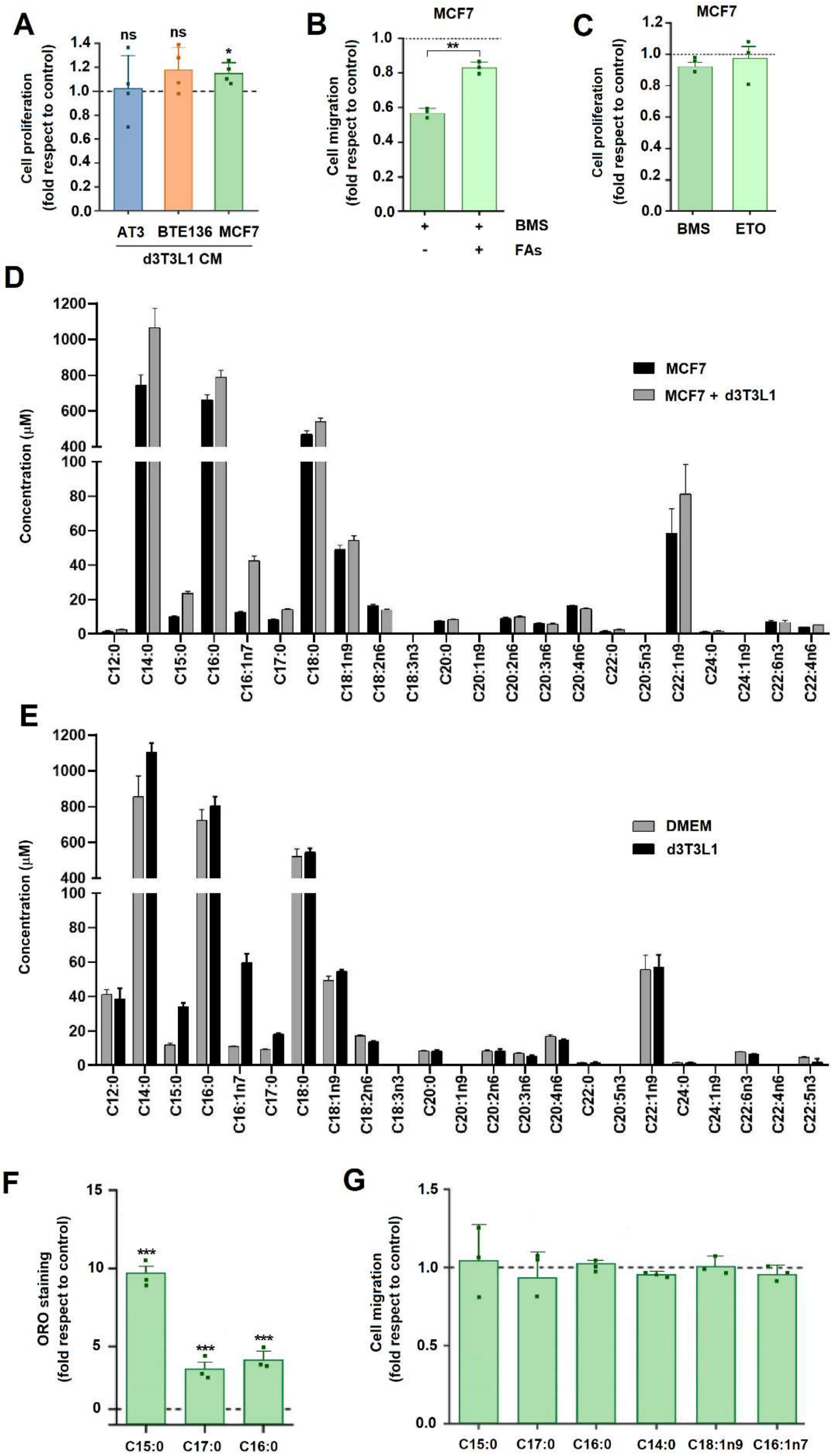

Suppl. Figure 3. **FAs do not promote tumor cell migration**. AT3, BTE136, and MCF7 cells were cultured with adipocytes conditioned medium (CM) (**A**) or treated with

Etomoxir (ETO, 30 μM) or BMS (20 μM) for 3 days (**C**). In A, proliferation was assessed by Crystal Violet staining. **B**, MCF7 cell migration was assessed as indicated in Methods in the presence of BMS (20 μM) and a mixture of FAs (C15:0, C17:0, C16:0, all 50 μM) for 48 h. **D-E**, the FA content of conditioned media from control MCF7, MCF7 co-cultured with differentiated 3T3L1 for 2 days, differentiated 3T3L1 cells or control DMEM was analyzed using GC-MS (see Suppl. Methods). The average of three independent experiments is shown. **F**, MCF7 cells were treated with the indicated FA (50 μM) for 2 days. Lipid accumulation was assessed using ORO staining and quantified after elution with isopropanol. **G**, MCF7 cell migration was measured after adding the indicated FA for 2 days. In A-C and F-G, the control corresponds to untreated MCF7 cells. ns, not significant; *, $p < 0.05$; **, $p < 0.01$; ***, $p < 0.001$.

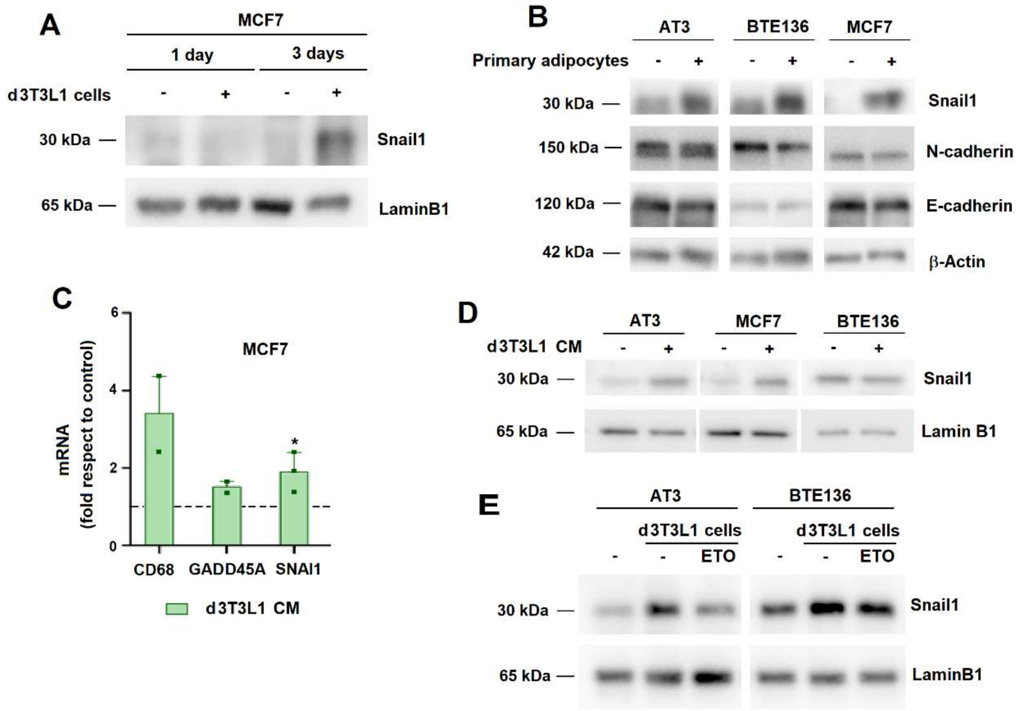

Suppl. Figure 4. **3T3L1 adipocytes increase Snail1 expression in tumor cells**. The indicated cells were co-cultured with differentiated 3T3L1 (d3T3L1) (**A, E**), with CM derived from these cells (**C, D**) or with primary adipocytes (**B**) for 3 days. When indicated, Etomoxir (ETO, 30 μM) was added. In C, the control corresponds to untreated MCF7 cells; the average ± SEM of three experiments (SNAI1), or ± the range of two experiments (CD68, GADD45A) is shown. *, $p < 0.05$.

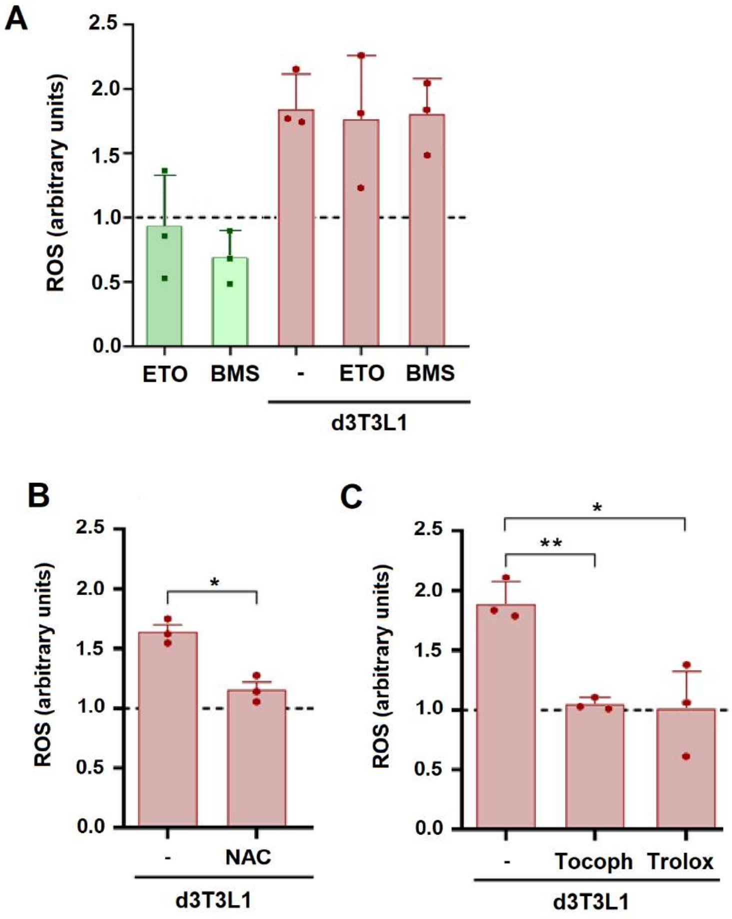

Suppl. Figure 5. **Co-culture with 3T3L1 adipocytes increases ROS production in MCF7 cells**. ROS production was assessed in MCF7 cells co-cultured with adipocytes for 3 days. When indicated, NAC (1 mM), Tocopherol (10 μM), Trolox (10 μM), Etomoxir (ETO, 30 μM) or BMS (20 μM) were added. The average ± SEM of three independent experiments is shown. *, $p < 0.05$; **, $p < 0.01$.

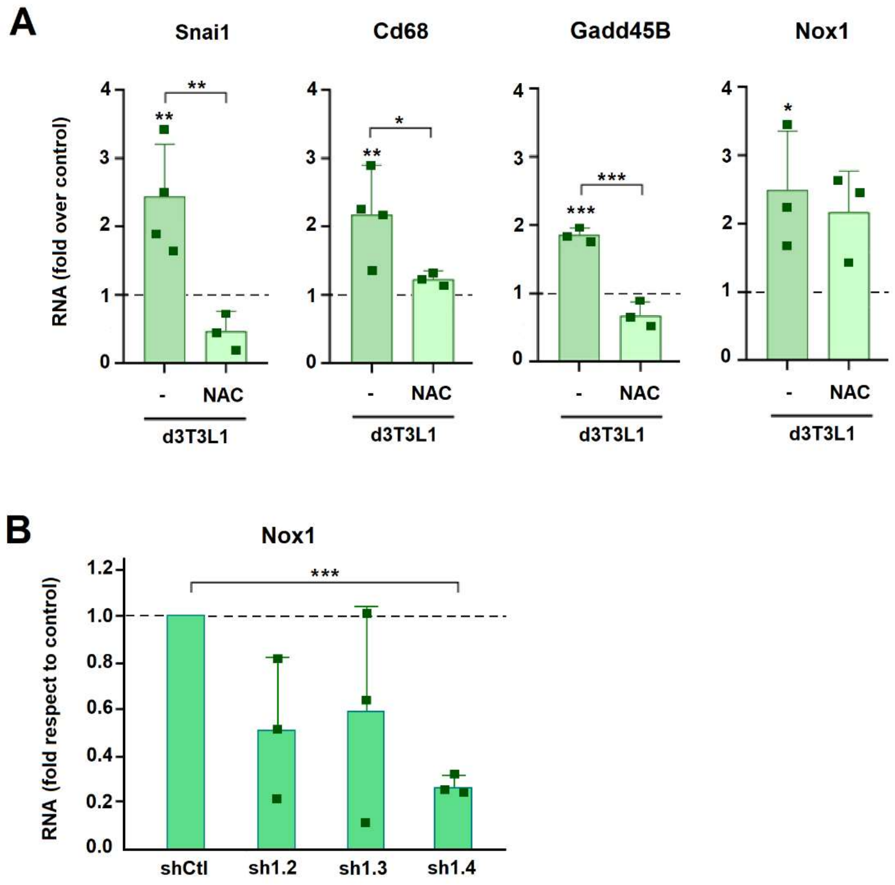

Suppl. Figure 6. **Co-culture with 3T3L1 adipocytes up-regulates mesenchymal gene expression in AT3 cells**. Levels of mentioned genes were determined by RT-PCR in AT3 cells co-cultured with adipocytes for 3 days (**A**) or in control conditions (**B**). When indicated, NAC (1 mM) was added. In B, the effect on Nox1 expression of the different Nox1 shRNAs transfected in AT3 was assessed and referred to cells transfected with shRNA control. The average ± SEM of three independent experiments is shown. *, $p < 0.05$; **, $p < 0.01$; ***, $p < 0.001$.

Gene	Oligonucleotides
**Murine**	
Cebpa	Fw: 5’-TTCGGGTCGCTGGATCTCTA-3’<br>Rv: 5’-TCAAGGAGAAACCACCACGG-3’
Cd68	Fw: 5’-GGGGCTCTTGGGAACTACAC-3’<br>Rv: 5’-GTACCGTCACAACCTCCCTG-3’
Cdh1	Fw: 5’-TTCAACCCAAGCACGTATCA-3’<br>Rv: 5’-ACGGTGTACACAGCTTTCCA-3’
Gadd45b	Fw: 5’-TAGAGGAACGCTGAGACCCA-3’<br>Rv: 5’-TCCCAGAAGGTATCACGGGT-3’
Hprt	Fw: 5’-GGCCAGACTTTGTTGGATTTG-3’<br>Rv: 5’-TGCGCTCATCTTAGGCTTTGT-3’
Lipe	Fw: 5’-GGAGCTCCAGTCGGAAGAGG-3’<br>Rv: 5’-GTCTTCTGCGAGTGTCACCA-3’
Nox1	Fw: 5’-CTCCAGCCTATCTCATCCTGAG-3’<br>Rv: 5’-AGTGGCAATCACTCCAGTAAGGC-3’
Rdh9	Fw: 5’-TGACCATCCCTCCCTGAAAAC-3’<br>Rv: 5’-GACTCACCAACCTAGCAGCA-3’
Slc2a4	Fw: 5’-ATCCGGAACCTGGAGGGGCC-3’<br>Rv: 5’-TGGCTCAGCTGCAGCACCAC-3’
Snai1	Fw: 5’-GCGCCCGTCGTCCTTCTCGTC-3’<br>Rv: 5’-CTTCCGCGACTGGGGGTCCT-3’
Pparg	Fw: 5’-GGCTGCAGCGCTAAATTCTT-3’<br>Rv: 5’-TGCGAGTGGTCTTCCATCAC-3’
**Human**	
CA9	Fw: 5´-ATCCACGTGGTTCACCTCAG-3´<br>Rv: 5´CGATTTCTTCCAAGGCACAC-3´
CD68	Fw. 5’-GGATTCACCAGTTCTGCCCA-3’<br>Rv: 5’-CCGCCATGTAGCTCAGGTAG-3’
CDH1	Fw: 5’-GAACGCATTGCCACATACAC-3’<br>Rv: 5’-ATTCGGGCTTGTTGTCATTC-3’
ENO2	Fw: 5´- TCCCACTGATCCTTCCCGAT-3´<br>Rev: 5´-TGACCTTGAGCAGCAGACAG-3´
GADD45A	Fw. 5’-CACTGTCGGGGTGTACGAAG-3’<br>Rv: 5’-GTTGATGTCGTTCTCGCAGC-3’
HPRT	Fw: 5’-GGCCAGACTTTGTTGGATTTG-3’<br>Rv: 5’-TGCGCTCATCTTAGGCTTTGT-3’
MYC	Fw: 5’-AACACACAACGTCTTGGAGC-3’<br>Rv: 5’-GCACAAGAGTTCCGTAGCTG-3’
NOX1	Fw: 5’-ACTCTTGGGGTAGGTGTGTG-3’<br>Rv: 5’-GGAGAGAATGGAGGCAAGGG-3’
NOX5	Fw: 5´-GGTGGGTGACTCAGCAGTT-3´<br>Rv: 5´-CAGCACCCCACTCTGTACCTG-3´
NOXA1	Fw: 5’-CTCGATGCAGAGACAGAGGTCG-3’<br>Rv: 5’-AGGAGCCTGTTTGCCAACTTGC-3’

SNAI1	Fw: 5'-GTGCCTCGACCACTATGCC-3'<br>Rv: 5'-GCTGCTGGAAGGTAAACTCTGG-3'

Suppl. Table 1. Oligonucleotides used in the RT-PCR assays.

Antibody	Host	Dilution	Reference	Supplier
β-Actin	Rabbit	1:10000	ab8227	Abcam
C/EBPα	Rabbit	1:1000	D56F10	Cell Signaling
Cytokeratin 14	Rabbit	1:1000	ab181595	Abcam
E-cadherin	Mouse	1:2000	610182	Transduction Labs
Fibronectin	Rabbit	1:10000	ab2413	Abcam
Glut4	Rabbit	1:1000	ab33780	Abcam
Lamin B1	Rabbit	1:2000	ab16048	Abcam
N-cadherin	Mouse	1:1000	610920	Transduction Labs
PPARɣ	Rabbit	1:1000	C26H12	Cell Signaling
p-Stat3 (Tyr705)	Rabbit	1:1000	9145S	Cell Signaling
Snail1	Rabbit	1:1000	SN9H2	Cell Signaling
Stat3	Rabbit	1:1000	D3Z2G	Cell Signaling

Suppl. Table 2. Primary antibodies used for Western blot analysis.
